# The Role of Inflammatory Mediators in the Development of Gastrointestinal Motility Disorders

**DOI:** 10.3390/ijms23136917

**Published:** 2022-06-22

**Authors:** Tibor Docsa, Adám Sipos, Charles S. Cox, Karen Uray

**Affiliations:** 1 Department of Medical Chemistry, Faculty of Medicine, University of Debrecen, 4032 Debrecen, Hungary; tdocsa@med.unideb.hu (T.D.); siposadam1102@gmail.com (A.S.); 2 Department of Pediatric Surgery, University of Texas Health Science Center at Houston McGovern Medical School, Houston, TX 77204, USA; charles.s.cox@uth.tmc.edu

**Keywords:** ileus, feeding intolerance, inflammation, cytokines, gastrointestinal motility

## Abstract

Feeding intolerance and the development of ileus is a common complication affecting critically ill, surgical, and trauma patients, resulting in prolonged intensive care unit and hospital stays, increased infectious complications, a higher rate of hospital readmission, and higher medical care costs. Medical treatment for ileus is ineffective and many of the available prokinetic drugs have serious side effects that limit their use. Despite the large number of patients affected and the consequences of ileus, little progress has been made in identifying new drug targets for the treatment of ileus. Inflammatory mediators play a critical role in the development of ileus, but surprisingly little is known about the direct effects of inflammatory mediators on cells of the gastrointestinal tract, and many of the studies are conflicting. Understanding the effects of inflammatory cytokines/chemokines on the development of ileus will facilitate the early identification of patients who will develop ileus and the identification of new drug targets to treat ileus. Thus, herein, we review the published literature concerning the effects of inflammatory mediators on gastrointestinal motility.

## 1. Introduction

Feeding intolerance and the development of ileus negatively impact critically ill, surgery, and trauma patients [1,2,3]. In this review, we define feeding intolerance as a delay in reaching feeding goals, and ileus is defined as the slowing or cessation of gastrointestinal motility (without mechanical blockage of the gastrointestinal tract), leading to feeding intolerance. We will not discuss the various clinical definitions of feeding intolerance and ileus, which are crucial in facilitating the development of better therapies for treating ileus and feeding intolerance; however, several good reviews and attempts at classifying at least post-operative ileus (POI) have been published recently [4,5,6,7].

As many as 60% of critically ill patients experience gastrointestinal motility disorders, which often lead to feeding intolerance [8]. A recent meta-analysis by Blaser et al. [3] shows that more than a third of intensive care patients suffer from some degree of feeding intolerance. After colorectal surgery, the incidence of prolonged POI ranges from 11% to 59% depending on how POI was defined, even when patients were subjected to enhanced recovery programs after surgery that were aimed at improving gastrointestinal function [9,10,11]. This demonstrates not only the high incidence of ileus but also the lack of effective treatments for ileus. The reported incidence of ileus in non-abdominal surgeries is usually lower but varies widely (2–18%) depending on the type of surgery [12,13,14]; however, considering the number of surgery patients, even 2% incidence still accounts for a large number of patients. We recently showed that one-third of moderate to severe trauma patients develop feeding intolerance [2]. This study included all trauma patients (blunt, penetrating, and burn injuries) requiring at least 3 days of intensive care. Only 40% of patients with feeding intolerance in our study had documented ileus (radiographic evidence), suggesting that ileus is under-diagnosed, at least in this patient group [2]. Critically ill, trauma, and surgery patients represent overlapping patient populations that account for a majority of hospitalized patients. The overall incidence of ileus in these patient populations ranges from 2% to 60%, representing a very large patient population. Despite the large number of patients affected by ileus, only a handful of new drugs have been developed to treat ileus in the last few decades.

Increased adverse outcomes are associated with feeding intolerance and ileus [11]. Blaser et al. demonstrated an increased mortality association with feeding intolerance [2,3]. We showed that patients with ileus are significantly more likely to be readmitted to the hospital within a year after the initial trauma [2]. In addition, patients with ileus are more likely to experience infectious complications like sepsis, urinary tract infections, and wound infections [2,9]. An increased incidence of deep vein thrombosis has also been associated with ileus [15]. Overall, these consequences of ileus result in longer hospital and intensive care unit stays, increased utilization of resources, and increased economic burden [2,14,16,17]. Furthermore, patients with ileus experience a significant reduction in quality of life, even 3 months after surgery [18].

Our understanding of the incidence, risk factors, and complications associated with ileus has improved in the last several decades. Clearly, the incidence of ileus is very high, affecting a large number of patients. Yet, surprisingly little progress in developing effective pharmacologic therapies to treat ileus and feeding intolerance has been made over the last several decades and the existing drugs have limited effectiveness and significant deleterious side effects. Treatment with erythromycin or metoclopramide did not shorten hospital and intensive care unit stays or lessen gastrointestinal symptoms related to ileus in hospitalized trauma patients [2]. In a 2008 Cochrane Review of drugs to treat POI, erythromycin (used to treat ileus in the US) was shown to be ineffective for the treatment of ileus and the effects were inconsistent or insufficient evidence was available to recommend any other drugs, including serotonin agonists (cisapride), dopamine antagonists (metoclopramide), beta-blockers (propranolol), vasopressin, or cholecystokinin-like drugs (cerulatide) [19]. In a more recent review by Sommer et al., the authors concluded that although preventative methods have advanced, there are no evidence-based effective treatments for ileus once it develops [20]. Furthermore, several drugs to treat ileus have significant deleterious side effects that limit their use [19]. Cisapride, a serotonin receptor (5-HT4) agonist, was pulled from the US market due to the danger of serious cardiac arrhythmias. The data supporting the use of plucalopride, another serotonin receptor agonist selective 5-HT4 receptor), are limited (level 1B) and the high rate of side effects may limit its use [21]. While Alvimopan has shown positive results in a meta-analysis [22], the Food and Drug Administration issued a black box warning for Alvimopan due to increased cardiovascular risk, allowing only in-hospital short-term (≤15 doses; ~2 weeks) treatment with this drug. Due to the lack of effective and safe therapies, ileus remains a significant clinical problem and there is a dire need for the identification of new drug targets for treating ileus. Understanding the mechanism by which ileus develops is crucial in identifying new drug targets for treatment.

The diagnosis of ileus is made based on symptoms, including abdominal distension, abdominal pain, constipation, and increased gastric residual volumes. These symptoms occur when the gut is already distended. However, early diagnosis of ileus is crucial in identifying which patients should be treated for ileus before the gut becomes distended and further injured. Increased stretch decreases myosin light chain phosphorylation via decreased MYPT1 phosphorylation in primary human intestinal smooth muscle cells [23]. Furthermore, conditioned media from macrophages subjected to increased stretch decreases the motility of the small intestine [24]. These studies indicate that distension of the gut wall alone can lead to smooth muscle dysfunction, which compounds the development of ileus. Thus, identifying early markers of ileus is crucial in preventing intestinal injury, which exacerbates and furthers the progression of dysfunction associated with ileus. Furthermore, when developing new prokinetics, identifying the appropriate patient population for drug trials depends on understanding which patients are likely to develop ileus. Therefore, understanding early markers of ileus/feeding intolerance is crucial. Specific inflammatory mediators or a combination of inflammatory mediators may be viable biomarkers for the early detection of ileus. For example, C-X-C motif chemokine ligand 1 (CXCL1), also known as GroA, is upregulated within the first 24 h after hospital admission in trauma patients who develop ileus compared with CXCL1 levels in trauma patients who do not develop ileus [24].

Plenty of evidence from both experimental and clinical studies suggests that inflammation contributes to the development of ileus and ileus coincides with the increased release of a variety of cytokines in both humans and animal models [25,26]. Inflammatory mediators play a critical role in the development of ileus via the activation of resident macrophages, the subsequent recruitment of neutrophils into the smooth muscle, and the activation of T helper cells, which propagate the spread of motility dysfunction along the GI tract [25,27,28,29,30]. However, the actual causative agent(s) of slowed gastrointestinal motility is unclear. Surprisingly, the effects of individual inflammatory mediators on intestinal motility have not been widely explored and the published studies are inconsistent or conflicting. The purpose of this review is to summarize the known effects of inflammatory mediators on intestinal motility. We focus, in particular, on the direct effects of inflammatory mediators on GI motility and highlight the conflicts in the published literature. Understanding which inflammatory cytokines/chemokines contribute to the development of ileus will facilitate the early identification of patients who will develop ileus and the identification of new drug targets to treat ileus. Thus, herein, we review the published literature concerning the effects of inflammatory mediators on gastrointestinal motility.

## 2. The Role of Inflammation in the Development of Ileus

As mentioned above, inflammatory mediators, including cytokines and chemokines, play an important role in the development of ileus. Laparotomy and handling of the gut during abdominal surgery or exploratory laparotomies in surgical and trauma patients activate macrophages in the gut wall [25,30]. Systemic inflammation in trauma patients induces intestinal edema, which distends the intestinal wall and may also activate resident macrophages in the intestinal wall [24]. Increased mechanical manipulation of macrophages increases the release of inflammatory mediators, including CXCL1 and IL-1β. The local inflammatory response induced by macrophage activation upregulates adhesion molecules, leading to the recruitment of leukocytes into the intestinal wall [29,31]. This mechanism of ileus is supported by data in human surgical patients that show that macrophages are activated in the intestinal wall in response to abdominal surgery [31]. The activation of macrophages and the recruitment of leukocytes result in the release of a variety of cytokines/chemokines and other inflammatory mediators. Mast cell degranulation also increases in response to intestinal manipulation and contributes to the development of ileus [32,33,34].

Although there are currently no effective drugs to treat ileus and no drugs targeting the inflammatory pathways contributing to ileus, several interventions may prevent ileus by preventing or attenuating inflammation in the gut. Several reviews and meta-analyses demonstrated that early enteral feeding reduces the incidence of ileus; however, not all studies supported the effects of early enteral feeding [7,35,36]. Interestingly, early enteral feeding may reduce mast cell activation and degranulation to attenuate the development of POI [33]. Feeding with lipid-rich enteral nutrition before surgery may also reduce mast cell degranulation and the release of cytokines into the peritoneum in a rodent (Sprague–Dawley rats) model of ileus [37].

## 3. Presence of Cytokine/Chemokine Receptors on Cells of the Gastrointestinal Tract

For cytokines/chemokines to have direct effects on gut motility, receptors need to be present on the surface of effector cells. While there is ample evidence that cytokines have effects on gastrointestinal motility, often the particular cell type mediating the response is unknown. Understanding cell-type specific responses is crucial in designing new treatment regimens to treat motility disorders. Thus, we describe the documented presence (either at the protein or mRNA level) of receptors on three cell types in the gastrointestinal tract that are crucial for contractile activity and motility: smooth muscle cells, enteric neurons, and interstitial cells of Cajal (ICCs). We include only direct evidence for the presence of receptors at the mRNA or protein levels, but there is often indirect evidence for the presence of receptors in the gastrointestinal tract, including knockout studies and receptor antagonist studies. Surprisingly, many gaps exist where we were unable to find direct documented evidence of receptors on specific cell types in the gut. Table 1 shows the confirmed expression of receptors for inflammatory mediators in the small and large intestine at the mRNA or protein levels by PCR, Western blotting, or immunohistochemistry (IHC).

### 3.1. Interleukin-1 Receptors

The interleukin-1 receptor family has 10 transmembrane proteins consisting of ligand binding members, accessory proteins, inhibitors, and several family members with unknown functions [57]. Only IL-1R1 and IL-1R2 bind IL-1α and IL-1β, but only IL-1R2 has a short intracellular domain that does not initiate signaling [58]. IL-1R3 acts as an accessory chain for IL-1R and is required for signaling, but it does not bind to IL-1α or β. Thus, we only focus on IL-1R1 since this chain is required for IL-1α and β signaling. Using IHC, Gougeon et al. showed that IL-1R1 was expressed in circular and longitudinal smooth muscle cells of the intestine and enteric neurons in the myenteric plexus in Sprague–Dawley rats [38]. Stoffels et al. also detected IL-1R1 in the myenteric plexus [40]. Stoffels did not observe the co-expression of IL-1R1 with CD117 (c-KIT), indicating that the ICCs in the small intestine do not express IL-1R1 [40]. Zhang et al. demonstrated increased IL-1R receptor mRNA levels in New Zealand rabbit colonic smooth muscle cells after colitis was induced with TNBS [39].

### 3.2. Interleukin-4 Receptors

IL-4 signaling requires both IL-4 receptor alpha (IL-4Rα) and a second receptor unit, the gamma common chain (γc) or IL-13Rα1 [59]. The IL-4Rα subunit first binds to IL-4, then recruits the second chain to the complex to initiate intracellular signaling [59]. Akiho et al. demonstrated IL-4Rα mRNA expression in dispersed longitudinal smooth muscle cells from the jejunum of C57Bl/6 mice and NIH Swiss mice through RT-PCR [42,43]. IL-4Rα mRNA was also detected in the enteric neurons and smooth muscle cells of the colon and small intestine of BALB/c mice [41]. The expression of IL-4Rα was higher in the colonic myenteric plexus compared to the intestinal myenteric plexus; however, the smooth muscle cell expression of IL-4Rα was similar in the two regions [41].

### 3.3. Interleukin-6 Receptors

IL-6 signals through the IL-6R. IL-6R forms a heterodimer with gp130, which transduces the IL-6 signal and initiates intracellular signaling [60]. IL-6R protein was observed in the colonic submucosal and myenteric plexus by IHC in both Sprague–Dawley and Wistar Kyoto rats [44,47]. Zhang et al. also showed that IL-6R is expressed in colonic smooth muscle cells and the myenteric plexus in Sprague–Dawley rats and expression, at both the mRNA and protein levels, increases after exposure to chronic unpredictable mild stress (e.g., cold swimming, cage tilting, heat stress, etc.) [46]. In addition, Deng et al. demonstrated the expression of IL-6R in colonic ICCs within the smooth muscle and the myenteric plexus using IHC in C57Bl/6 mice [45].

### 3.4. Interleukin-13 Receptors

IL-13 binds to both IL-13Rα1 and IL-13Rα2. The IL-13Rα1 chain binds IL-13 with low affinity and subsequently forms a heterodimer with IL-4Rα (the type 2 IL-4R) to induce downstream signaling [61]. Interestingly, the recruitment of IL-4Rα to the IL-13/IL-13α1 complex increases the affinity of IL-13 to IL-13α1 [62]. The binding of IL-13 to IL-13Rα2 does not induce downstream signaling and signaling via this receptor is poorly understood at present [61]. IL-13Rα1 mRNA was detected in the colon and small intestines of BALB/c mice in enteric neurons and smooth muscle cells [41]. In contrast, IL-13α2 (mRNA) was only expressed in smooth muscle cells and not in the myenteric plexus of BALB/c mice [41]; the expression of IL-13α2 was higher in the colon compared to the small intestine.

### 3.5. Interleukin-17 Receptors

IL-17 mainly signals through the IL-17RA/IL-17RC receptor heterodimers [63]. Binding of IL-17 to the first receptor alters the affinity and specificity of the receptor to favor heterodimer formation [64]. Both IL-17RA and IL-17RC mRNAs were detected in intestinal smooth muscle sheets in Sprague–Dawley rats [48]. Although the mucosal and submucosal layers were removed, it was unclear if smooth muscle sheets were separated from the myenteric plexus in this study. In BALB/c mice, constitutive IL-17RA mRNA expression was detected in intestinal smooth muscle cells [49]. In contrast, IL-17RC was not detected in intestinal smooth muscle in an immunohistochemical study of normal human tissues [65].

### 3.6. CXCR1 and CXCR2 Receptors

Interleukin-8 (also known as CXCL8), which can exist as a monomer or dimer, signals through the G-protein coupled receptors, CXCR1 and CXCR2 [66,67]. CXCL1 also signals through CXCR2 receptors. CXCR1 expression is widespread and is expressed in the gastrointestinal tract; in smooth muscle cells of other organs, including airway and vascular smooth muscle cells; and in neural cells, including dorsal root ganglia [68,69,70]. Although CXCR2 is expressed in the intestine [71], we could find no publications confirming the expression of CXCR1 and CXCR2 in specific cell types in the large or small intestine.

### 3.7. TGF-β Receptors

TGF-β exerts most of its effects through three receptors: TGFR-1, TGFR-2, and TGFR-3. TGFR-1 and -2 are serine/threonine kinases. TGF-β binds to either TGFR-2 directly or binds to TGFR-3, which presents TGF-β to TGFR-2. Once activated, TGFR-2 binds and activates TGFR-1 via transphosphorylation [72]. Akiho and colleagues demonstrated the expression of TGFR-2 at the mRNA level in dispersed jejunal longitudinal smooth muscle cells isolated from C56Bl/6 and NIH Swiss mice [43,52]. Hagl et al. demonstrated the mRNA expression of TGFR-1-3 in Wistar rat myenteric plexus and intestinal smooth muscle cells [53]. In humans, Hagl demonstrated mRNA expression in the myenteric plexus and tunica muscularis [53].

### 3.8. Interferon-γ Receptors

Interferon gamma (IFN-γ) signals by forming a dimer and binding to an IFNγ receptor (IFNGR) heterodimer. The IFNGR is composed of the high- and low-affinity IFNGR 1 and 2, respectively [73]. Although IFN-γ receptors are thought to be widely expressed, little information was available concerning the cell type-specific expression of IFN-γ receptors in the large and small intestines. Akiho and colleagues showed the expression of IFNGR at the mRNA level in dispersed jejunal longitudinal smooth muscle cells isolated from C56Bl/6 mice [52].

### 3.9. TNF-α Receptors

TNF signaling is mainly mediated through TNF receptor 1 (TNFR1) and TNFR2. Trimeric TNF binds to three receptors (forming homotrimers) to form a functional signaling unit [74]. Gougeon et al. showed that TNFR1 was expressed in the circular and longitudinal smooth muscle cells of the colon and enteric neurons in the myenteric plexus in Sprague–Dawley rats using IHC [38]. TNFR1 and R2 are both expressed on dispersed human colonic circular smooth muscle cells, as demonstrated by IHC [55]. Chandrasekharan et al. demonstrated that functional TNFR1 and R2 are expressed in cultured enteric neurons [56]. Interestingly, Eisenman et al. showed that TNFR1, but not TNFR2, was expressed in cultured gastric ICCs from BALB/c mice [75].

## 4. Cytokine Sources in the Gastrointestinal Tract

The gastrointestinal tract has the most immune cells in the body. The gastrointestinal-associated lymphoid tissue (GALT), which makes up 70% of the immune system by weight, can respond and discriminate between harmful and harmless antigens [76,77]. In addition to the Peyer’s Patches, other immune cells reside in the intestinal wall, including resident macrophage and dendritic cells. Immune cells, such as neutrophils, can be recruited to the gastrointestinal tract in response to the activation of immune cells. All of the immune system cells are sources of inflammatory cytokines and chemokines. Even the mesenteric lymph can be a source of inflammatory mediators. TNF-α significantly increased in the mesenteric lymph within 60 min after LPS treatment in a sepsis rat model (Sprague–Dawley) [78]. These phenomena have been well described in the literature and will not be described here. Instead, we focus on the secretion of cytokines by non-immune system cells, including smooth muscle cells, enteric neurons, and ICCs. Enteric glial cells are crucial in the crosstalk between different cell types affecting gastrointestinal motility and secrete a variety of cytokines and other inflammatory mediators [79]. Interestingly, the release of IL-1βα and β from mesenteric glial cells seems to require the microbiome [26].

Khan et al. demonstrated that primary intestinal smooth muscle cells, isolated from rats, secrete IL-6 in response to IL-1 [80]. Van Assche et al. also demonstrated that IL-1 treatment of isolated intestinal smooth muscle cells induces the secretion of IL-6 and this effect was blocked by IL-1 receptor antagonists [81]. More recently, the secretion of TNF-α and IL-1β by colonic smooth muscle cells from BALB/c mice in response to LPS was demonstrated by Al-Dwairi et al. [82]. Co-treatment with metformin attenuated the effects of LPS on cytokine secretion in this model. In human circular smooth muscle cells, the secretion of IL-6, IL-1β, and IL-8 can be induced by IL-1β, TNF-α, and/or IFN-γ [55,83]. LPS was shown to induce the secretion of CXCL1 and IL-8 from smooth muscle cells via the toll-like receptor 4 [84]. Interestingly, static stretch alone induced the secretion of IL-1β, and mechanical stretch greatly enhanced the LPS-induced secretion of IL-1β in cultured intestinal smooth muscle cells isolated from Lewis rats [85]. Thus, intestinal distension during the development of ileus may increase the secretion of inflammatory cytokines to intensify the development of ileus.

Cultured enteric neuronal cells have been shown to secrete IL-6, TNF-β, and MCP-1 when stimulated with LPS [86,87]. TGF-β 1–3 mRNAs were detected in the enteric nervous system and smooth muscle layers in both rats and humans, indicating that these cell types are capable of secreting TGB-β [53]. Overall, a variety of different signals can induce the secretion of cytokines and chemokines from the non-immune cells of the gastrointestinal tract.

## 5. Effects of Cytokines/Chemokines on Gastrointestinal Motility

Although this review focuses on the role of inflammatory mediators in the development of ileus, we describe the role of inflammatory mediator-induced changes in gastrointestinal motility in other diseases as well. Inflammation has been shown to both increase and decrease gastrointestinal motility. The intestinal contractile activity was increased in mice in response to *Trichinella spiralis*-induced inflammation [88], while intestinal motility was decreased by inflammation induced by surgical manipulation, 2,4,6-trinitrobenzensulfonic acid (TNBS) treatment, and peritonitis [30,89,90]. Understanding the specific effects of individual inflammatory mediators is necessary to understand the conflicting effects of different inflammatory conditions on gastrointestinal motility.

There is some consensus that ileus is primarily mediated by a TH1-mediated immune response, although there is some evidence suggesting the TH2 cells may also contribute to ileus [28,91,92]. This fits in with the trend that TH1 cytokines generally decrease intestinal contractile responses while TH2 cytokines, such as IL-4 and IL-13, increase gastrointestinal motility. Thus, IL-4 and IL-13 are not likely to be involved in the development of ileus. Despite this overall trend for TH1 and TH2 cytokines, the responses to several cytokines are controversial; both an increased and decreased contractile activity in response to these cytokines are reported. To begin to discern the reasons for discrepancies in the literature, we have included information on the models, species, strains, cell types, and cytokine doses. A summary of the effects of inflammatory mediators on gastrointestinal motility is shown in Table 2. Cytokines with conflicting effects are shown in bold.

### 5.1. IL-1

IL-1β has been shown to both increase and decrease gastrointestinal activity depending on the section of the GI tract, the cell type, timing, and the disease model. Of note, the short (<24 h) and long-term (>24 h) effects of IL-1 may be different. A number of investigators have demonstrated the inhibitory effects of IL-1β on intestinal motility. Aubé et al. showed that IL-1β (90 and 150 min; 10–50 ng/mL) inhibits intestinal contractility in Wistar rat jejunal and colonic strips [93]. In this study, IL-1β inhibited acetylcholine-induced—but not basal—contractions and required new protein synthesis. Furthermore, the effects of IL-1β were mediated by the myenteric plexus and were independent of nitric oxide [93]. In Sprague–Dawley rats, an IL-1β receptor antagonist blocked the inhibitory effects of LPS, indicating that IL-1β inhibited the motility of the rat small intestine in an IL-1 receptor-dependent manner [94,95]. Short-term IL-1β treatment suppressed acetylcholine release from the longitudinal intestinal smooth muscle myenteric plexus in rats (60–90 min; 10 ng/mL) [96].

Ohama et al. showed that long-term (3 days) treatment of rat (Wistar, male) intestinal smooth muscle tissue with IL-1β inhibits agonist-induced contractile activity via the downregulation of CPI17, an endogenous inhibitor of myosin light chain phosphatase, and decreased inhibitory phosphorylation of MYPT1, the myosin targeting subunit of myosin light chain phosphatase [97,98]. Long-term IL-1β treatment (2 days; 10 ng/mL) also inhibited acetylcholine-induced colonic smooth muscle cell contractions via the downregulation of CPI-17 in rabbits [99]. Interestingly, IL-1β (5 days, 10 ng/mL) downregulates CPI17 in IL-1β, but not in TNF-α knockout mice (C57BL/6), suggesting that the effects of IL-1β are mediated through TNF-α. IL-1β also prolongs the calcium response to stimuli, such as serotonin and ATP, in the myenteric plexus [100]. In other cell types, IL-1β induces RhoA activation [101]. However, Hu et al. showed that Il-1β did not affect RhoA expression or acetylcholine-induced ROCK activation in colonic smooth muscle cells [99].

In contrast to the findings described above, Nalli et al. showed that the treatment of mouse colonic longitudinal smooth muscle cells with IL-1β enhanced the cholinergic induction of MLCK activity and MLC phosphorylation [102]. IL-1β enhancement of the cholinergic response was mediated by the inhibition of AMPK activity and suppression of inhibitory MLCK phosphorylation. In a guinea pig myenteric plexus preparation, Kelles et al. showed that IL-1β excited myenteric neurons in a receptor-dependent tetrodotoxin-resistant manner [103]. Interestingly, Il-1β affected both excitatory and inhibitory motor neurons. Thus, IL-1β could conceivably both increase and decrease intestinal motility, which may explain some of the disparate effects of IL-1β.

In the stomach, Suto et al. showed that the intravenous or intracisternal injection of IL-1β decreased gastric emptying in rats, likely through central-mediated mechanisms [104]. In a rat model, surgically-induced delayed gastric emptying was reversed by an IL-1β receptor antagonist, supporting the role of IL-1β in suppressing gastric emptying [105]. In humans, higher serum levels of IL-1β and TNF-a were associated with decreased gastric emptying and survival [106].

IL-1β appears to play a role in a number of disease models. In a gut manipulation model of ileus, IL-1α and β inhibition prevented the development of POI [40]. In a *Trichinella spiralis* infection model, in addition to intestinal motility, an intraperitoneal injection of IL-1β in rats decreased gastric motility, while IL-1 receptor antagonism blocked the decreased gastric motility induced by lipopolysaccharide (LPS) [107].

IL-1β has mostly been shown to depress gastrointestinal contractile activity in animal models; however, the results in humans are less consistent. Interleukin-1β has been shown to inhibit contractile activity in human colonic circular smooth muscle from patients with ulcerative colitis [108,109]. IL-1β has also been shown to negatively correlate with gastric emptying [110]. However, IL-1β was barely detectable and not significantly different in patients with prolonged POI after colorectal surgery [111]. In contrast, in another study, IL-1β, along with IL-6 and procalcitonin, was higher in patients with prolonged POI compared to patients who did not develop ileus following surgery for colorectal carcinoma [112].

For the most part, evidence from animal models supports the conclusion that IL-1β inhibits gastrointestinal motility. Although the role of IL-1β in the development of ileus in patients is less clear, IL-1β likely depresses gastrointestinal motility in humans as well. The development of ileus is likely to be multifaceted and IL-1β may be one of several inflammatory mediators that contribute to the development of ileus. Alternatively, IL-1β may be upregulated during the development of inflammation leading to ileus, but may not directly affect gastrointestinal motility.

### 5.2. IL-4

According to published studies, IL-4 increases gastrointestinal motility. In the late 1990s, Goldhill et al. showed that short-term treatment with IL-4 increased the intestinal contractile response to cholinergic nerve stimulation, but not to acetylcholine, indicating that the response was likely mediated by enteric neurons (BALB/c mice) [113]. The treatment of longitudinal smooth muscle from the small intestines of BALB/c mice with IL-4 for 1 week resulted in an enhanced contractile response to electrical field stimulation, which could be blocked by tetrodotoxin, confirming the role of enteric neurons in the IL-4 response [114]. On the other hand, genetic ablation of STAT6 did not affect this enhanced contractile activity induced by IL-4 in the mouse model [114]. Increased IL-4 expression (adenoviral-mediated expression) at the serosal surface of the small intestine in C57Bl/6 mice also elicited increased carbachol-induced contractile activity [115]. Interestingly, while prophylactic treatment of BALB/c mice with IL-4 blocking antibodies prevented allergy-induced diarrhea, post-treatment did not prevent the diarrhea [116]. In animal models, the consensus is that IL-4 induces hyper-responsiveness to cholinergic agonists in the small intestine and this hyper-responsiveness is mediated by the enteric nervous system.

Akiho et al. showed that IL-4 was significantly increased in the muscularis layers from human ileal samples of Crohn’s patients [117]. In cultured human intestinal smooth muscle cells, IL-4 enhanced carbachol-induced contractions, and the inhibition of STAT6 blocked this enhancement [117]. IL-4 levels did not change significantly in IBS patients compared to normal controls [118]. However, IL-4 may play a significant role in allergic diarrhea, parasitic expulsion, and Crohn’s disease.

Of note, there were several differences in the effects of IL-4 between animals and humans. First, while the IL-4-induced hyper-contractile activity seems to be predominantly mediated by the enteric nervous system in animal models, human smooth muscle cells responded to IL-4 directly, according to the Akiho study [117]. Furthermore, while the effects of IL-4 on intestinal contractile activity (induced by electrical field stimulation) were not affected by the amelioration of STAT6 in the mouse model of parasitic infection, STAT6 inhibition blocked the enhanced carbachol-induced contractile response in human intestinal smooth muscle cells [114]. Overall, it appears that IL-4 increases intestinal motility, but the mechanism is unclear.

### 5.3. IL-6

Published studies show conflicting reports concerning the effects of IL-6 on gastrointestinal motility. Several studies suggest that IL-6 inhibits gastrointestinal motility. IL-6 inhibition prevented the decreased gastrointestinal motility in a sepsis mouse model (cecal ligation and puncture) [119]. In a murine (C3H/HeOuJ) endotoxin-induced ileus model, IL-6 inhibition (IL-6R antibody) prevented late ileus development [120]. Increased IL-6 was associated with reduced colonic and jejunal motility in multiple studies, including multi-organ failure and shock rat models, respectively—although direct effects were not measured [121,122].

On the other hand, IL-6 was shown to increase colonic contractions in rats [46,123,124]. IL-6 was also shown to increase colonic motility in a rat model of irritable bowel syndrome and inhibition of IL-6 normalized stress-induced defecation [124]. IL-6 treatment increased colonic contractility in non-diabetic rats, whereas inhibition of IL-6 signaling decreased colonic motility in diabetic (streptozotocin-treated) rats [125]. An intraperitoneal injection of IL-6 in rats did not affect GI motility [107].

In humans, IL-6 was associated with delayed gastric emptying [126]. IL-6 was also associated with delayed gut motility in ulcerative colitis patients [127]. Interestingly, IL-6 was significantly elevated in premature infants with confirmed necrotizing inflammation, but not infants with feeding intolerance only [128]. Furthermore, the presence of IL-6 did not predict prolonged POI in colorectal surgery patients [111]. In the esophagus, IL-6 was associated with decreased contractile activity [129].

Overall, the results show that while IL-6 is often upregulated in models of ileus or feeding intolerance, the effects of IL-6 on gastrointestinal motility are unclear and may be context- and species-dependent. The association of IL-6 with altered gastrointestinal motility in humans is unclear.

### 5.4. IL-8

Plattner et al. demonstrated that IL-8 enhanced the cholinergic contractile response, but not spontaneous contractile activity, in the ileum of Wistar rats in a dose-dependent and time-dependent (>60 min) manner [130]. The response was not affected by TTX, indicating that the effect was not neurally mediated. However, the enhanced contractile activity was inhibited by cycloheximide, indicating a requirement for new protein synthesis, and required the presence of the mucosa, indicating that the response is probably not a direct effect of IL-8 on the smooth muscle or ICCs. IL-8 also increased the colonic contractile activity in SD rats [124]. In addition, neutralizing IL-8 antibodies blocked the enhancement of colonic contractile activity elicited by plasma from IBS patients [124]. However, in contrast to the findings in the small intestine, TTX blocked the effects of IL-8 on colonic contractile activity, indicating that the effects of IL-8 were mediated by enteric neurons in the colon.

While increased IL-8 was associated with increased lengths of hospital stays, IL-8 did not correlate with the length of bowel recovery after colorectal surgery [131]. IL-8 also did not correlate with gastrointestinal symptoms in patients with IBS [132]. However, IL-8, along with other cytokines, was elevated in ulcerative colitis patients with IBS-like symptoms, including constipation, diarrhea, and abdominal pain [133]. Although IL-8 was associated with enhanced immune activity in IBS patients, serum IL-8 levels did not correlate with stool frequency or transit time [134]. These studies indicate no clear correlation between IL-8 and gastrointestinal symptoms. On the other hand, IL-8 and TNF-α were positively correlated with abdominal symptoms in IBS-D patients [135]. In a study comparing plasma levels of inflammatory mediators in patients with obstructive, vascular, or paralytic ileus to normal individuals, the combination of IL-8 and TNF-α were significantly correlated with vascular ileus [136]. Interestingly, polymorphisms in the IL-8 promoter are associated with several types of infectious diarrhea [137,138]; however, it is unclear if these are associated with changes in gastrointestinal motility. Overall, the link between IL-8 and altered gastrointestinal motility in clinical diseases is unclear.

### 5.5. IL-13

IL-13 increases gastrointestinal contractile activity in both animal models and human cells. Treatment of longitudinal smooth muscle from the small intestines of BALB/c mice with IL-13 for 1 week resulted in an enhanced contractile response to electrical field stimulation, which could be blocked by tetrodotoxin [114]. This enhanced response was absent in STAT6 knockout mice. Treatment with IL-13 also enhanced the response to acetylcholine [114]. The increased spontaneous contractions induced by a 7-day IL-13 treatment of BALB/c mice were absent in IL-13 knockout mice [139]. Interestingly, in an intestinal anaphylaxis mouse model (BALB/c), diarrhea developed in wild-type mice, but not in IL-13 knockout mice, indicating a role of IL-13 in allergy-induced diarrhea [116]. IL-13 also plays a role in intestinal parasitic infection. An antagonist of IL-13 prevents *N. brasiliensis* expulsion in a STAT6-dependent manner, indicating that IL-13 plays a role in host protection against nematodes [140]. IL-13 is upregulated in *T. spiralis* infection and likely plays a role in the *T. spiralis*-induced hypercontractility [141].

Similar to the animal models, treatment of primary human intestinal smooth muscle cells with IL-13 enhanced carbachol-induced contractions in a STAT6-dependent manner [117]. Interestingly, an SNP in the promoter region of the IL-13 gene, resulting in increased IL-13 expression, is associated with IBS [142]. However, the SNP does not associate with diarrhea or constipation predominant phenotypes and may, therefore, not be related to changes in gastrointestinal motility. In the esophagus, mucosally-expressed IL-13 is associated with increased esophageal contractile activity [129]. Although there is little data in clinical studies concerning the direct effects of IL-13 on gastrointestinal motility, the few studies that do exist are generally in agreement with the animal studies that show that IL-13 increases gastrointestinal motility.

### 5.6. IL-17

Both increased activity and decreased contractile activity in response to IL-17 were reported. In a post-inflammation model of GI motility dysfunction (BALB/c), treatment (1–3 days) with IL-17a was associated with increased agonist-induced contractile activity and increased myosin light chain phosphorylation. This hypermotility was attenuated by treatment with IL-17RFc, an antibody that blocks the interaction of IL-17a with its receptor [143]. Fu et al. also showed that the treatment of mouse (male Inbred NIH mice) intestinal smooth muscle strips with IL-17 (2 d, 10 ng/mL) increased the agonist-induced (10^−6^ M acetylcholine) contractile activity [144]. However, in a *Giardia* infection model, knockdown of IL-17a did not prevent post-infectious hypermotility [145]. In a *T. spiralis*-infected mouse (c57Bl/6) model, I-17 expression is increased and the administration of recombinant IL-17 increased intestinal the contractile activity, which was attenuated by ROCK inhibition, but not COX-2 and STAT-6 pathway inhibitors [50].

In contrast to the reports above, Mori et al. found that in rats (Sprague–Dawley), the treatment of intestinal tissue with IL-17a (12 h–3 d treatment) suppressed agonist-induced contractile activity and this effect was reversed with a nitric oxide synthase inhibitor [48]. In a mouse model of sepsis (BALB/c), gene expression profiling showed that the IL-17 pathway was associated with decreased contraction amplitude and frequency (24 and 48 h) and the blockade of IL-17A (IP injection with IL-17A antibody) improved motility in a cecal ligation and puncture model of sepsis [146]. Buchholz et al. also found that IL-17 was associated with late rodent ileus (24 h, C57BL/6) [147]. In a traumatic brain injury rat (SD) model, increased IL-17, along with IL-1α and β, was associated with slowed intestinal transit [148].

Very little data are available concerning IL-17 and ileus in patients. However, decreases in IL-17 were associated with a shorter gastric emptying time in preterm infants treated with *L. Reuteri* [149]. In contrast, treatment with IL-17 did not affect agonist-induced human colonic longitudinal muscle contractile activity when measured ex vivo [150].

### 5.7. TNF-α

Studies consistently show that TNF-α suppresses colonic contractile activity. In a mouse (C57Bl/6) TNBS-induced colitis model, TNF-α was significantly increased in the smooth muscle layers of the colon (2 days after treatment) and TNBS-induced colonic motility dysfunction was attenuated in TNF-α deficient mice compared to wild-type mice [151]. Treatment of mouse colonic tissue for 3 days with TNF-α suppressed carbachol-induced contractions [151]. Similar results were obtained in a DSS mouse model (129 3/SvImJ) of colitis; TNF-α inhibition (etanercept or XPro1595) improved colonic contractile activity [56]. In a rat (Wistar) TNBS-induced colitis model, Ohama et al. showed that TNF-α inhibits the expression of CPI-17, an endogenous inhibitor of myosin light chain phosphorylation [97]. In addition to the colitis models, TNF-α was also shown to be inhibitory in other models of gastrointestinal dysfunction. In a rat (Wistar) multi-organ failure model, TNF-α levels were negatively correlated with colonic contractile activity [121]. In addition to the suppression of contractile activity, TNF-α also suppressed forskolin-induced relaxation in mouse (C57Bl/6J) colonic smooth muscle cells [152]. Of note, changes in TNF expression do not coincide with the onset of ileus in a mouse (C57Bl/6) gut manipulation model of ileus (in this case, ileus is attributed to TH1 cytokines) [28]. In contrast to the inhibitory effects of TNF-α in the colon described above, Al-Shboul et al. showed that the treatment of mouse (C57BL/6J) colonic longitudinal smooth muscle cells with TNF-α augmented the response to acetylcholine [153].

Although most studies demonstrate inhibitory effects of TNF-α in the colon, the effects of TNF-α in other parts of the gastrointestinal tract are less clear. An intraperitoneal injection of TNF-α did not affect gastric motility in a rat model (Sprague–Dawley) [107]. Interestingly, TNF-α secreted from M1 macrophages reduced gastric ICC levels; this mechanism may be partially responsible for the delayed gastric emptying in diabetes [75].

Investigations into the effects of TNF-α on small intestinal motility are inconsistent. In mouse ileal smooth muscle (C57BL/6J), IL-1α inhibited intestinal contractile activity via TNF-α-mediated downregulation of CPI-17 [97]. However, Ford et al. found that treatment of human intestinal smooth muscle cells with TNF-α did not affect tonic contractions [154]. Furthermore, treatment of rats (Sprague–Dawley) with TNF-α (0.25–0.35 mg/kg for 5 min and function measured 24 h later) did not significantly affect intestinal transit, although there was a synergistic effect of TNF-α and morphine on intestinal permeability [155]. Of note, the effects of TNF-α in the colon required longer treatments and may be dependent on new protein synthesis.

In human gastrointestinal tissue studies, TNF-α effects were inconsistent. Pazdrak et al. demonstrated that 24-h treatment of human colonic muscle strips inhibited contractile activity induced by acetylcholine [156]. Shi and Sarna also showed that TNF-α treatment for 24 h suppressed agonist-induced (acetylcholine) contractile activity in human colonic smooth muscle strips [157]. However, Safdari et al. showed that the direct treatment of human colonic tissue (from resection after malignancy) with TNF-α (10 ng/mL for 20 hrs) did not affect maximal agonist-induced (acetylcholine) contraction or acetylcholine EC_50_ [150]. As mentioned in the previous paragraph, Ford et al. also showed no effects from TNF-α on tonic contractions in human intestinal smooth muscle cells [154].

TNF-α was associated with depressed gastrointestinal motility in clinical studies. In a prospective study of early enteral nutrition in critically ill patients, TNF-α was significantly higher in patients who did not receive early enteral nutrition due to feeding intolerance, although TNF-α levels were similar before the start of early enteral nutrition [158]. TNF-α was also significantly higher in patients who developed prolonged POI after colorectal surgery compared to patients who did not develop ileus [112]. TNF-α was significantly higher in ulcerative colitis patients with prolonged orocecal transit time [127]. TNF-α levels did not correlate with changes in gastric emptying and increased gastric emptying in response to prokinetics did not alter TNF-α levels [159]. Interestingly, in a study of IBS patients, TNF-α was significantly higher in diarrhea-predominant—but not constipation predominant— IBS patients relative to healthy patients [132]. In a meta-analysis, the serum levels of TNF-α were elevated in all IBS subtypes [160]. In contrast to the small and large bowel effects, TNF-α was associated with increased contractile activity in the esophagus [129].

In summary, most animal and human studies show that TNF-α suppresses contractile activity in the small intestine and colon, but there are discrepancies.

### 5.8. IFN-γ

Several investigators have shown that IFN-γ suppresses human intestinal smooth muscle cell contractions and responses to cholinergic agonists [154]. Using a gel contraction assay, Ford et al. showed that treatment of human intestinal smooth muscle cells with IFN-γ (as low as 62.5 units/mL, dose-dependent effect) results in an inhibition of gel contraction within 1 day after treatment, but also a decrease in smooth muscle cell proliferation [154]. Intestinal manipulation has been shown to induce IFN-γ expression in the small intestine and colon of mice (c57Bl/6), and knockdown of IFN-γ attenuated the reduced intestinal and colonic transit induced by gut manipulation [28]. Pre-incubation with IFN-γ decreased the ligand affinity of muscarinic receptors and decreased carbachol-induced longitudinal muscle contractility [52]. In a mouse model (AKR), parasite infection was associated with colonic hypocontractility and increased IFN-γ levels [161]. Antihelminthic treatment decreased IFN-γ levels and improved colonic contractile activity in this model, even though myeloperoxidase activity remained high.

As mentioned above, INF-γ inhibited contractile activity in human intestinal smooth muscle cells. However, the role of IFN-γ in clinical diseases is less clear. IFN-γ and IL-4 levels were measured in human ileal samples from patients with Crohn’s disease and compared to healthy individuals. IFN-γ did not change significantly in the muscularis layers in diseased sections, while IL-4 increased significantly [117]. IFN-γ levels tended to decrease in IBS patients compared to healthy patients [52,134].

### 5.9. TGF-α

Most animal and cellular studies show that TGF-β increased gastrointestinal motility. Treatment of mouse (C57Bl/6) jejunal longitudinal smooth muscle cells with TGF-β increased the response to carbachol; the ED50 for carbachol was decreased significantly [52]. Interestingly, TGF-β significantly increased the agonist affinity of muscarinic receptors in the intestinal smooth muscle in this study [52]. Using a gel contraction assay, Moore-Olufemi et al. showed that TGF-β1-3 increases human intestinal smooth muscle cell contractile activity [162]. Furthermore, the intraperitoneal administration of TGF-β3 shortened the intestinal transit time in rats (Sprague–Dawley) [162]. The infection of mice (NIH Swiss) with *T. spiralis* resulted in a prolonged elevation in TGF-β expression (up to at least 35 days after infection), which was thought to maintain the hypercontractility induced by Th2 cytokines, resulting in the subsequent upregulation of COX-2 and PGE-2 in smooth muscle cells [43]. The effects of *T. spiralis* on TGF-β expression were confirmed by other investigators [50]. However, Steel et al. showed that TGF-β induces the activation of Th17 cells and the subsequent secretion of IL-17, which causes hypercontractility after infection [50]. In contrast to these studies, colonic contractile activity was decreased in a transgenic mouse model (TβRIIΔk-fib TG mice) in which TGF-β signaling is increased [163]. However, this is attributed to fibrosis development in the colon [163].

TGF-β has been implicated in several gastrointestinal diseases. The inhibition of TGF-β signaling by SMAD7 has been demonstrated in IBD patients [164]. TGF-β3 was significantly increased (and TGF-β1 tended to increase, *p* = 0.06) in the intestinal smooth muscle layers of infants with gastroschisis compared to premature infant controls [162]. Loss-of-function mutations in the *TGFB1* gene are associated with severe, early onset IBD [165]. Although TGF-β signaling has been implicated in a number of gastrointestinal diseases, the impact of TGF-β on gastrointestinal motility is often unclear in these diseases; TGF-β may modulate the immune response or fibrosis development rather than directly affecting gastrointestinal motility.

### 5.10. MCP-1 (Monocyte Chemoattractant Protein 1, CCL2)

Animal studies show that MCP-1 has an inhibitory effect on intestinal motility. Decreased MCP-1, along with IL-1β, TNF-α, and IL-6, was associated with improved GI transit in a gut manipulation model of ileus (mice) after a number of different treatments, including the transcutaneous stimulation of the auricular branch of the vagus nerve (C57BL/6 mice, 24-h time point), mangiferin treatment (Swiss mice, 24 h time point), and 5-HT3 inhibition (Balb/c, 3 h) [166,167,168]. Interestingly, Sonnier showed that MCP-1 and macrophage-derived chemokine (MDC/CCL22) were both secreted into the lumen in an endotoxemia mouse model (IP injection of LPS) and oral gavage with MDC resulted in a significant slowing of intestinal transit [169]. However, treatment with MCP-1 (C57Bl/6 mice) did not decrease intestinal transit (measured 6 hrs after oral gavage with MCP-1) [169]. In conflict with this study, the inhibition of MCP-1 with neutralizing antibodies partially attenuated the decreased carbachol-induced colonic contractions induced by colitis (TNBS) in a rat model (Sprague–Dawley) [170,171]. MCP-1 is thought to be released by macrophages in response to endotoxin [172].

In humans, higher MCP-1 and RANTES levels (measured at 24 h) were associated with a prolonged time for the restoration of bowel function (>5 days) in colorectal surgery patients [131].

### 5.11. Other Inflammatory Mmediators and Signaling Pathways

Investigations into the role of inflammatory mediators in the development of ileus have been somewhat biased, in that most investigators check a handful of classic inflammatory mediators, such as IL6 and TNF-α, without examining other inflammatory mediators. We reviewed the literature for other inflammatory mediators that are potentially involved in the development of ileus and/or slowed gastrointestinal motility.

The role of CXCL1 in the gut has been mostly studied in the context of inflammatory or infectious diseases. However, CXCL1 is upregulated in animal models of ileus and inhibits intestinal contractile activity in mice and rats [24,147,173]. In addition, CXCL1 was significantly higher in trauma patients who developed ileus compared to trauma patients with no slowed motility [24].

In a gut manipulation model of ileus in mice (C57BL/6), IL-10 deficiency attenuated the development of ileus [174]. Interestingly, Stein et al. demonstrated that IL-10 loss impeded neutrophil migration to traumatized tissue; thus, IL-10 probably did not have a direct effect on intestinal motility, but may be active in the inflammatory injury to the smooth muscle [174].

Several studies have implicated the role of Jak/Stat signaling in the development of ileus. In an intestinal manipulation murine model of ileus, JAK1 was upregulated and the inhibition of JAK (administered preoperatively) attenuated the decreased intestinal motility induced by gut manipulation [175]. Consistent with this study, the inhibition of STAT3 also attenuated depressed intestinal motility in an intestinal edema rat model of ileus [176].

## 6. Conclusions

In general, TH1 cytokines inhibit gastrointestinal motility and TH2 cytokines increase gastrointestinal motility. However, there are many exceptions to this generalized theory. Furthermore, the reported effects of individual cytokines and chemokines are sometimes conflicting. The conflicts may be due to different species, different models, spontaneous versus agonist-induced contractile activity measurements, age, and context dependency—including the role of mechanotransduction and the presence of other inflammatory markers [177,178,179]. The importance of context is demonstrated in Docsa et al., where conditioned media from activated macrophages inhibits intestinal contractile activity in tissue collected after laparotomy, but not in tissue collected from naive animals [24]. Inflammatory reactions are complex, involving a variety of cytokines. Although most laboratory studies examine responses to a single cytokine, changes in a single cytokine do not occur in vivo. Furthermore, the half-lives of inflammatory mediators are different and may be altered in different cellular milieu. Different immune responses to gut manipulation and LPS in rats and mice may also explain some of the differences in cytokine responses [180]. Within the murine models, different strains have different immune response deficiencies that could change the response to cytokines. Many studies demonstrate the role of inflammation in the development of ileus and other gastrointestinal motility disorders; however, the mechanism by which inflammatory mediators cause changes in intestinal motility is poorly understood and requires more investigation. To date, no drugs targeting the inflammatory pathways have been developed to treat or prevent ileus. Understanding the effects of inflammatory mediators on intestinal motility will facilitate the identification of common pathways that can be targeted to treat ileus.

## Figures and Tables

**Table 1:** Cell-specific receptor expression in the small and large intestines.

Receptor	Small Intestine			Colon			
	SMC	ICC	ENS	SMC	ICC	ENS	REF
IL-1R1	SD rats, protein	-	SD rats, protein; C57BL/6 mice, protein	NZ rabbit, mRNA	-	-	[38,39,40]
IL-4Rα	Balb/c mouse, mRNA; C57Bl/6 mice, mRNA; NIH Swiss mouse, mRNA	Balb/c mouse, mRNA	Balb/c mouse, mRNA	Balb/c mouse, mRNA	Balb/c mouse, mRNA	Balb/c mouse, mRNA	[41,42,43]
IL-6R	-	-	-	SD rats, mRNA and protein	C57Bl/6 mouse, protein	SD and WKY rats, protein; SD rats, mRNA and protein	[44,45,46,47]
IL-13RA1	Balb/c mouse, mRNA	Balb/c mouse, mRNA	Balb/c mouse, mRNA	Balb/c mouse, mRNA	Balb/c mouse, mRNA	Balb/c mouse, mRNA	[41]
IL-13RA2	Balb/c mouse, mRNA	-	-	Balb/c mouse, mRNA	-	-	[41]
IL-17RA	SD rat, mRNA; BABL/c mice, mRNA; C57Bl/6 mice, mRNA	-	-	-	-	-	[48,49,50]
IL-17RC	SD rat, mRNA	-	-	-	-	-	[48]
CXCR2	-	-	-	-	-	-	
TGFR-1	Human, protein; C57Bl/6 mice, mRNA; Wistar rats, mRNA; CD1 mice, protein		Wistar rats, mRNA;	human, mRNA		human, mRNA	[51,52,53,54]
TGFR-2	Human, protein; C57Bl/6 mouse, mRNA; NIH Swiss mouse, mRNA; Wistar rats, mRNA; CD1 mice, protein		Wistar rats, mRNA;	human, mRNA		human, mRNA	[43,51,52,53,54]
TGFR-3	Wistar rats, mRNA	-	Wistar rats, mRNA;	human, mRNA	-	human, mRNA	[53]
IFNGR1	C57Bl/6 mouse, mRNA	-	-	-	-	-	[52]
TNFR1	SD rat, protein	-	SD rat, protein; 129 mice, mRNA	Human, protein	-	-	[38,55,56]
TNFR2	-	-	129 mice, mRNA	Human, protein	-	-	[55,56]

CXCR2, CXC chemokine receptor; ENS, enteric nervous system; ICC, interstitial cells of Cajal; IL, interleukin; INFGR1, interferon-γ receptor 1; NIH, National Institutes of Health; NZ, New Zealand; SD, Sprague–Dawley; SMC, smooth muscle cell; TGFR, transforming growth factor receptor; TNFR, tumor necrosis factor receptor.

**Table 2:** The effects of inflammatory mediators on gastrointestinal motility.

Location	Effect	Inflammatory Mediator
Small intestine	↓ contractility	IL-1β, IL-6, **IL-17**, IFN-γ, CXCL-1
	↑ contractility	IL-4, IL-13, **IL-17**, TGF-α
	↓ acetyl choline-induced contractions	IL-1β, TNF-α
	↑ acetyl choline-induced i contractions	IL-4, IL-8, IL-13, IL-17, TGF-α
Colon	↓ colonic motility	**IL-1β**, **IL-6**, **TNF-α**, IFN-γ, TGF-α
	↑ colonic motility	**IL-1β**, **IL-6**, IL-8, **TNF-α**, MCP-1
	↑ acetyl choline-induced colonic contractions	IL-1β
Stomach	↓ gastric emptying	IL-1β, IL-6
	↑ gastric emptying	IL-17
Whole Animal	↑ POI	IL-1β, IL-6, IL-17, IL-10
	↓ GI transit	IL-6, IL-17, MCP-1
	↑ GI transit	TGF-α

↓ indicated downregulation and ↑ indicates upregulation; Bolding indicates cytokines with conflicting actions reported in the literature. CPI, C-kinase potentiated Protein phosphatase-1 Inhibitor; CXCL1, C-X-C motif chemokine 1; ENS, enteric nervous system; IL, interleukin; IFN, interferon; GI, gastrointestinal; MCP, monocyte chemoattractant protein; MLC, myosin light chain; MLCK, myosin light chain kinase; MYPT1, myosin phosphatase targeting subunit 1; POI, post-operative ileus; TGF, transforming growth factor; TNF, tumor necrosis factor.

## Data Availability

Not applicable.
